# Pretreatment but not subsequent coincubation with midazolam reduces the cytotoxicity of temozolomide in neuroblastoma cells

**DOI:** 10.1186/s12871-015-0135-4

**Published:** 2015-10-17

**Authors:** Sebastian Braun, Inge Bauer, Benedikt Pannen, Robert Werdehausen

**Affiliations:** Department of Anaesthesiology, University Hospital Düsseldorf, Moorenstr. 5, 40225 Düsseldorf, Germany

**Keywords:** Temozolomide, Midazolam, Cytotoxicity, Hormesis, Cell cycle

## Abstract

**Background:**

Temozolomide (TMZ) induces a G2/M cell cycle arrest and is used for treatment of paediatric tumours, especially neuroblastomas. Patients treated with TMZ frequently receive midazolam for sedation prior to surgery and other interventions. Previous studies suggested both cytoprotective and apoptosis-inducing properties of midazolam. Therefore, the impact of midazolam on TMZ-induced cytotoxicity was investigated *in vitro.*

**Methods:**

Human neuroblastoma cells were incubated with midazolam alone, as a pretreatment prior to incubation with TMZ or a coincubation of both. Cell viability and proliferation was analysed (XTT and BrdU assay) after 24 h and flowcytometric cell cycle analysis was performed after 24 and 48 h.

**Results:**

Midazolam alone increased cell viability at lower concentrations (2, 4, 8, 16 μM), whereas higher concentrations (128, 256, 512 μM) reduced cell viability. Pretreatment with midazolam 6 h prior to TMZ incubation reduced cytotoxic effects (IC_25_ 1005 ± 197 μM; IC_50_ 1676 ± 557 μM; *P* < 0.05) compared to incubation with TMZ alone (IC_25_ 449 ± 304 μM; IC_50_ 925 ± 196 μM) and reduced the antiproliferative effect of TMZ (1000 μM) by 43.9 % (*P* < 0.05). In contrast, cytotoxic effects of TMZ were increased (IC_75_ 1175 ± 221 μM vs. 2764 ± 307 μM; *P* < 0.05) when midazolam pretreatment was followed by coincubation of midazolam and TMZ. Cell cycle analysis revealed increased fractions of cells in G2/M phase after TMZ treatment (100 μM; 48 h), irrespective of midazolam pretreatment.

**Conclusion:**

Midazolam causes a hormetic dose–response relationship in human neuroblastoma cells. Pretreatment with midazolam reduces the cytotoxic and antiproliferative effects of TMZ without interfering with G2/M cell cycle arrest. In contrast, subsequent midazolam coincubation increases overall cytotoxicity.

## Background

In the past decade, the relevance of temozolomide (TMZ), a DNA-methylating agent, for the treatment of paediatric solid tumours, especially neuroblastomas, has been elucidated [1–6]. There is evidence, that even relapsed or refractory tumours respond to TMZ alone or in combination with other cytostatic agents [1–3, 7]. TMZ acts as a prodrug, which is spontaneously converted to the cytotoxic compound 5-(3-methyltriazen-1-yl) imidazole-4-carbozamide (MTIC) and alkylates guanine in genomic DNA at position *O*^6^ with subsequent impaired DNA-repair and G2/M arrest [8]. Reduction of the cytotoxic effect of TMZ by concomitant medication might reduce the effectiveness of anticancer regimen.

Midazolam is one of the most common sedatives for paediatric premedication [9, 10] and procedures that require temporary sedation (e.g. imaging) and has been reported recently the be among the 20 most often utilised medications in oncology patients associated with toxic side effects [11]. Midazolam has been shown to exert apoptosis-inducing properties, which are mediated by the mitochondrial pathway in neuroblastoma cells and primary rat neurons [12], while other modes of cytotoxicity were reported to be predominant in other human cell types [13]. Whereas high concentrations of midazolam (>50 μM) induce apoptosis in a large fraction of neuroblastoma cells and primary rat neurons, low concentrations of midazolam (5–10 μM) produce a slight increase of cell viability in primary rat neurons [12]. This effect has not been investigated in neuroblastoma cells so far. Thus, the influence of low concentrations of midazolam on cell viability in this cell line remains unknown. The increase of cell viability might counteract the cytotoxic effect of chemotherapeutics like TMZ and contribute to a reduced anticancer activity of these agents. Furthermore, no data are available, whether midazolam is able to modify the cell cycle of neuroblastoma cells, especially in regard to the TMZ-induced G2/M arrest.

We hypothesised, that midazolam at low concentrations increases cell viability, leading to a reduced toxicity of the anticancer agent TMZ by altering the cell cycle. A viability assay (XTT) was performed to evaluate the effect and toxicity of increasing concentrations of midazolam and TMZ. Additionally, a bromodeoxyuridine-based proliferation assay (BrdU) was used to estimate the effect of midazolam to the antiproliferative effect of temozolomide. The toxicity of TMZ (IC_25_, IC_50_ and IC_75_) after pretreatment and comedication with midazolam (16 μM) was investigated and cell cycle analysis was performed to elucidate the effect of midazolam on the primary mode-of-action of TMZ.

## Methods

### Materials and reagents

Roswell Park Memorial Institute 1640 medium (RPMI) with and without phenol red and trypsin/EDTA (0.05 %/0.02 %) were purchased from PAN-Biotech (Aidenbach, Germany). Penicillin and streptomycin were obtained from PAA Cell Culture Company (Cambridge, UK). NaCl, KCl, KH_2_PO_4_ and fetal calf serum were obtained from Merck (Darmstadt, Germany). Ethanol was obtained from Carl Roth GmbH (Karlsruhe, Germany). Na_2_HPO_4_, DMSO, EDTA, propidium iodide, phenazine methosulfate, TMZ and staurosporine were purchased from Sigma-Aldrich (St. Louis, MO, USA). Preservative-free midazolam was obtained from ratiopharm GmbH (Ulm, Germany). 2,3-Bis-(2-methoxy-4-nitro-5-sulfophenyl)-2H-tetrazolium-5-carboxanilide (XTT) sodium salt was obtained from AppliChem GmbH (Darmstadt, Germany).

### Cell culture

Human neuroblastoma cells (SHEP) (characterised in [14, 15]) were cultured in RPMI medium, supplemented with 10 % heat-inactivated fetal calf serum, 2 mM L-glutamine, 50 U mL^−1^ penicillin and 50 μg mL^−1^ streptomycin. Cells were cultured at 37 °C in a humidified 5 % carbon dioxide atmosphere.

### Cell treatment and experimental setup

Cell viability was assessed using the XTT assay after the following treatments: 1: increasing concentrations of midazolam for 24 h (0, 1, 2, 4, 8, 16, 32, 64, 128, 256, 512 μM); 2: increasing concentrations of TMZ for 24 h (control, 10, 30, 100, 300, 1000 and 2000 μM); 3: increasing concentrations of TMZ for 24 h after pretreatment with midazolam 16 μM for 6 h; 4: increasing concentrations of TMZ for 24 h after pretreatment with midazolam 16 μM for 6 h or pretreatment + coincubation for 24 h.

Cell cycle analysis using the Nicoletti assay [16] was performed after incubation with 1.: TMZ (0, 10, 100, 1000 μM) for 24 h, 2.: pretreatment with midazolam 16 μM for 6 h with subsequent incubation with TMZ (0, 10, 100, 1000 μM) for 24 h, 3.: TMZ (0, 10, 100, 1000 μM) for 48 h, 4.: pretreatment with midazolam 16 μM for 6 h with subsequent incubation with TMZ (0, 10, 100, 1000 μM) for 48 h.

### Analysis of cell viability

The XTT assay was used for measurement of cell viability. Briefly, XTT (2,3-Bis(2-methoxy-4-nitro-5-sulfonyl)-2H-tetrazolium-5-carboxanilide inner salt) is metabolised to a coloured formazan dye in vital cells by mitochondrial dehydrogenase. Due to the fact, that only vital cells are capable to metabolise XTT, the amount of produced formazan dye is indicative for the fraction of vital cells. To evaluate cell viability, 100 μl of cell suspension with a cell concentration of 10^5^ ml^−1^ per well were incubated overnight in a 96-well plate. Each well was washed with PBS to remove unattached cells and refilled with 200 μl colourless medium (RPMI without phenol red), supplemented with different concentrations of midazolam and TMZ alone or in combination. After 24 h, 100 μl cell culture supernatant were removed from each well and 50 μl of the dye solution were added, containing XTT (1 mg ml^−1^) and phenazine methosulfate (50 μM). After gentle mixing of the samples, the plate was incubated for 120 min at 37 ° C. Subsequently, samples were measured spectrophotometrically at 540 nm.

### Analysis of cell proliferation

A colorimetric immunoassay was used to quantify cell proliferation. Shortly, during DNA synthesis, proliferating cells integrate the thymidine analogue 5-bromo-2’-deoxyuridine (BrdU) instead of thymidine. Following denaturation, a peroxidase-conjugated antibody binds to newly incorporated BrdU. After a specific substrate reaction with tetramethylbenzidine, spectrophotometrical measurement of absorbance indicates the extent of newly synthesised DNA as a parameter of proliferation. In detail, 200 μl of a cell suspension containing 1x10^4^ ml^−1^ cells were incubated in each well of a 96-well plate overnight to ensure adherence and logarithmic growth. Subsequent incubation with the investigated substances was followed by fixing the cells and denaturation. Incubation with anti-BrdU-peroxidase conjugated specific antibodies to BrdU. After a washing step with 200 μl PBS and subsequent addition of 100 μl substrate solution containing tetramethylbenzidine, absorbance was measured at 370 nm and 492 nm (read-out absorbance = absorbance_370nm_ - absorbance_492nm_).

### Cell cycle analysis

Cell staining with propidium iodide allows quantifying the amount of cellular DNA, which indicates the phase of the cell cycle. After permeabilisation and staining, the quantity of cellular DNA is analysed by flow cytometric analysis, using the intensity of fluorescence of the stained DNA. Modifications of the cell cycle by drugs like TMZ are indicated by a change of the flow cytometric patterns [16].

For cell cycle analysis, 2 ml of cell suspension were incubated with a concentration of 0.5x10^5^ cells ml^−1^ overnight to reach adherence. Medium was replaced and supplemented with the investigated substance. After the defined time period, supernatant was harvested and adherent cells were detached with trypsin/EDTA 0.05 %/ 0.02 % at 37 °C and added to the supernatant. After centrifugation (1600 rpm, 5 min, 21 °C), the supernatant was removed and cells were washed with 1 ml of PBS. Subsequently, cells were incubated with 250 μl Nicoletti buffer (containing sodium citrate dihydrate 0.1 %, Triton X-100 0.1 % and propidium iodide 50 μg ml^−1^) and kept on ice until completion of flow cytometric analysis.

### Statistics

Results are expressed as means ± SD. All calculations were made with GraphPad Prism version 5.03 for Windows (GraphPad Software, San Diego, CA, USA). The values for drug concentrations leading to 25 %, 50 % or 75 % reduction of cell viability (IC_25_, IC_50_ and IC_75_, respectively) and the zero equivalent point (ZEP) were obtained from nonlinear regression analysis for bell-shaped dose–response relationships. Statistical analysis was performed by means of Student’s *t*-Test or one-way analysis of variance (ANOVA) with Bonferroni post hoc test. *P* < 0.05 was considered significant.

## Results

### Cell viability after incubation with different concentrations of midazolam

To evaluate the impact of midazolam on the viability of neuroblastoma cells, XTT assay was performed after incubation for 24 h with increasing concentrations of midazolam. In particular with regard to low concentrations of midazolam, small increments of concentrations were selected.

**Fig. 1:**
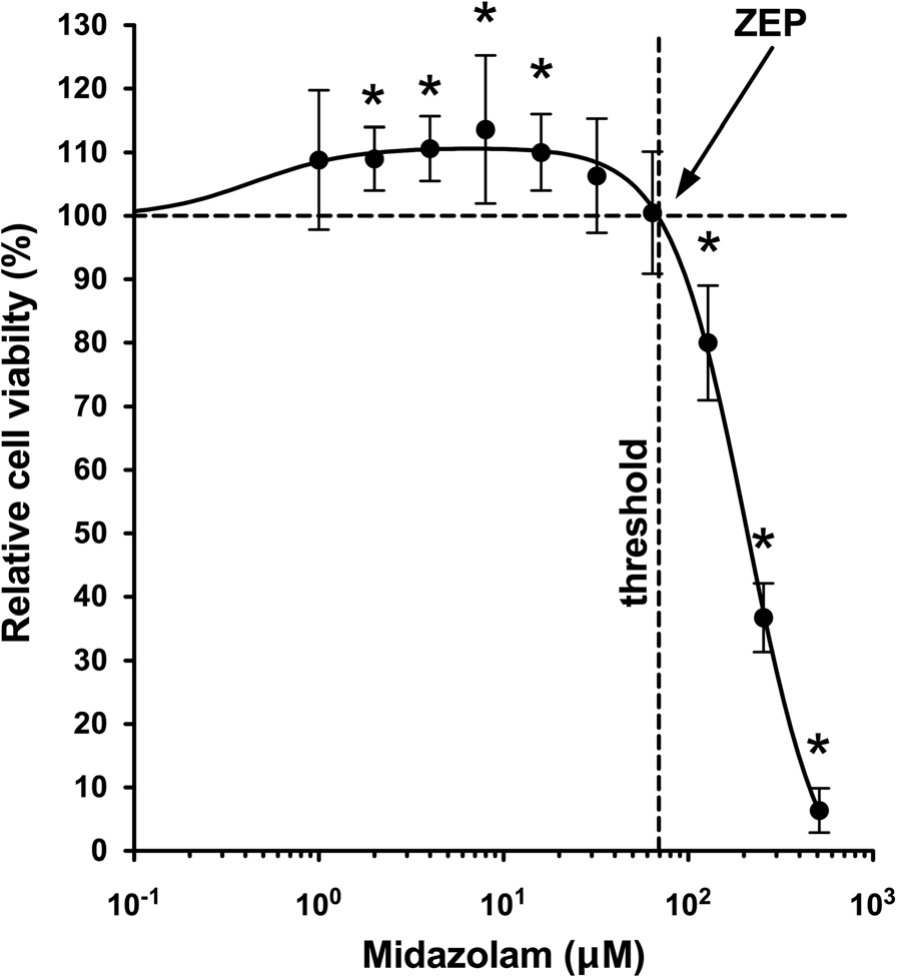
Midazolam-induced hormesis and toxicity in neuroblastoma (SHEP) cells. Cell viability of neuroblastoma (SHEP) cells was measured with the XTT assay to investigate cell viability after incubation for 24 h with increasing concentrations of midazolam. The "Zero Equivalent Point" (ZEP) describes the point of reversal of response and defines the concentration of midazolam, which is characterised by the transition from protective to toxic effects [17]. The ZEP of midazolam was 79.4 μM. A significant increase of cell viability in comparison to control was detected after incubation with 2, 4, 8, and 16 μM, whereas a significant decline was measured after incubation with 128, 256 and 512 μM midazolam (*n* = 8; * = *P* < 0.05 compared to control (0 μM midazolam); data are expressed as mean ± SD)

Midazolam increased cell viability in neuroblastoma cells at the lower concentrations used (2, 4, 8 and 16 μM), whereas higher concentrations (128, 256 and 512 μM) led to a profound decline of cell viability (Figure 1). Thus, midazolam caused a hormetic response with regard to cytotoxicity, characterised by a low-concentration stimulation and a high-concentration inhibition. The "Zero Equivalent Point" (ZEP) describes the point of reversal of response and defines the concentration of midazolam, which is characterised by the transition from protective to toxic effects [17]. The ZEP of midazolam was 79.4 μM. The IC_50_ of midazolam in the investigated neuroblastoma cell line was 226.9 ± 13.2 μM.

### Impact of pretreatment and pretreatment + coincubation of midazolam to the IC_25_, IC_50_ and IC_75_ of temozolomide

**Fig. 2:**
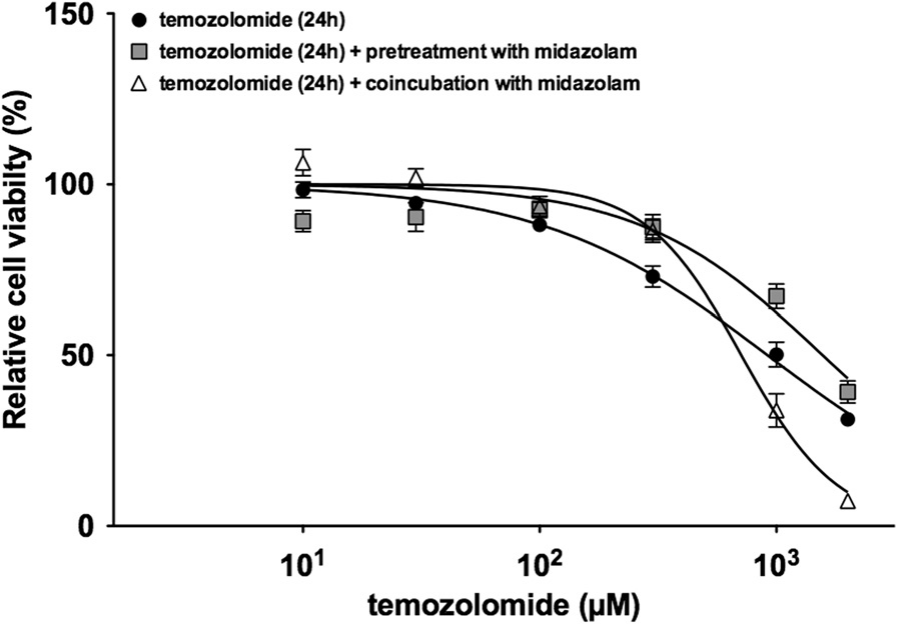
Effect of pretreatment and coincubation with midazolam to temozolomide induced toxicity. Cell viability of neuroblastoma (SHEP) cells was measured using the XTT assay after incubation with increasing concentrations of temozolomide (24 h). Black circles: relative cell viability after incubation with TMZ (10, 30, 100, 300, 1000, 2000 μM); greys squares: pretreatment with midazolam (16 μM) for 6 h with subsequent incubation with increasing concentrations of temozolomide; white triangles: pretreatment with midazolam (16 μM) followed by coincubation with temozolomide (24 h) (*n* = 5; data are expressed as mean ± SD)

**Fig. 3:**
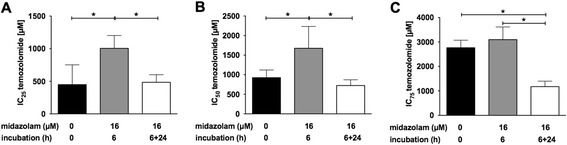
Midazolam modifies the toxicity of temozolomide. The IC_25_, IC_50_ and IC_75_ (Panel **a**, **b**, and **c**, respectively) were calculated with nonlinear regression analysis. Whereas pretreatment without subsequent coincubation increased the IC_25_ and IC_50_ of temozolomide, pretreatment followed by coincubation with midazolam did not alter the IC_25_/IC_50_ of temozolomide. The IC_75_ was significantly decreased after pretreatment followed by coincubation with midazolam, indicating an additive toxic effect (*n* = 5; data are expressed as mean ± SD; * = *P* < 0.05

The dose–response of TMZ was investigated without midazolam and after pretreatment (6 h prior to TMZ incubation) or pretreatment followed by coincubation (6 h prior to TMZ incubation and subsequent coincubation for 24 h) with midazolam (16 μM) (Fig. 2). Pretreatment with midazolam (16 μM) increased both the IC_25_ (1005 ± 197 μM vs. 449 ± 304 μM; *P* < 0.05, Fig. 3a) and IC_50_ (1676 ± 557 μM vs. 925 ± 196 μM; *P* < 0.05, Fig. 3b). In contrast, the IC_75_ of TMZ was unaffected by midazolam pretreatment (Fig. 3c).

Midazolam pretreatment followed by coincubation with TMZ did not change the IC_25_ and IC_50_ of TMZ, while the IC_75_ of TMZ was decreased significantly (IC_75_ 1175 ± 221 μM vs. 2764 ± 307 μM; *P* < 0.05), indicating an amplification of the TMZ-induced cytotoxicity (Fig. 3a-c).

### The influence of pretreatment and pretreatment + coincubation of midazolam to the antiproliferative effect of temozolomide

**Fig. 4:**
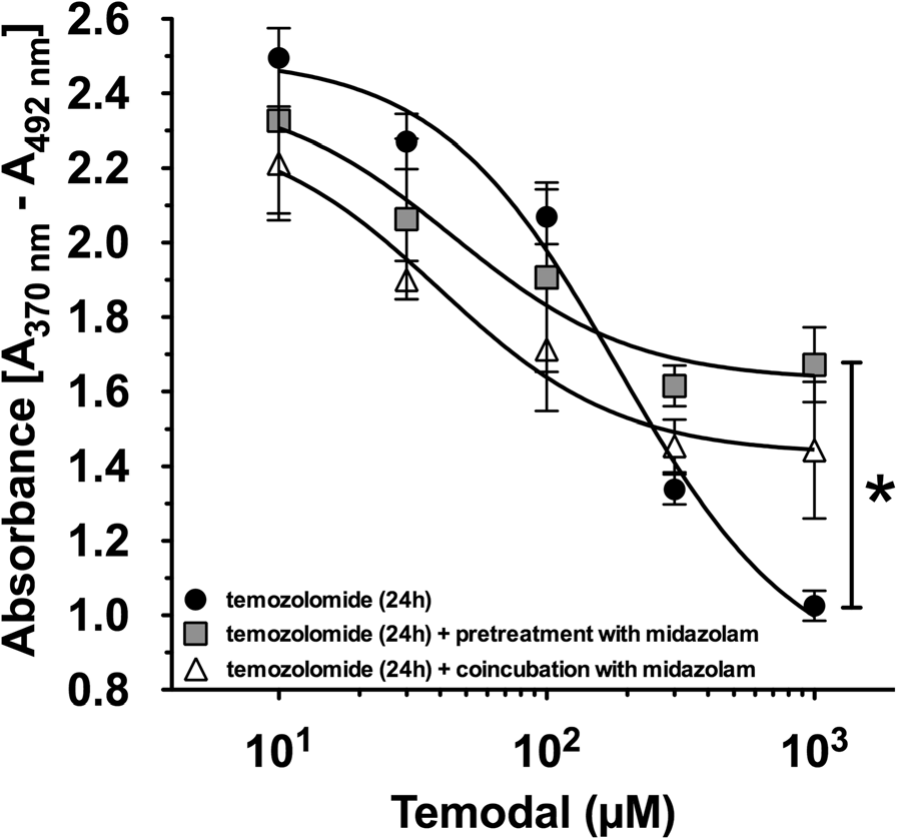
Pretreatment with midazolam reduces the antiproliferative effect of temozolomide. Proliferation of neuroblastoma (SHEP) cells was measured by the BrdU assay. In presence of temozolomide (TMZ) 1000 μM, pretreatment with midazolam enhanced cell proliferation, whereas coincubation with midazolam had no significant impact to the TMZ-induced antiproliferative effect (*n* = 3-4; data are expressed as mean ± SD; * = *P* < 0.05)

The proliferation rate of neuroblastoma (SHEP) cells was measured using the BrdU-assay after incubation with increasing concentrations of TMZ with and without midazolam. Pretreatment with midazolam 6 h prior to TMZ exposure significantly attenuated the antiproliferative effect of TMZ 1000 μM by 43.9 % (*P* < 0.05; Fig. 4), whereas midazolam pretreatment for 6 h with further coincubation for 24 h with TMZ did not alter the antiproliferative effect of TMZ (Fig. 4).

### Affection of the cell cycle after incubation with different concentrations of temozolomide with and without pretreatment of midazolam

**Fig. 5:**
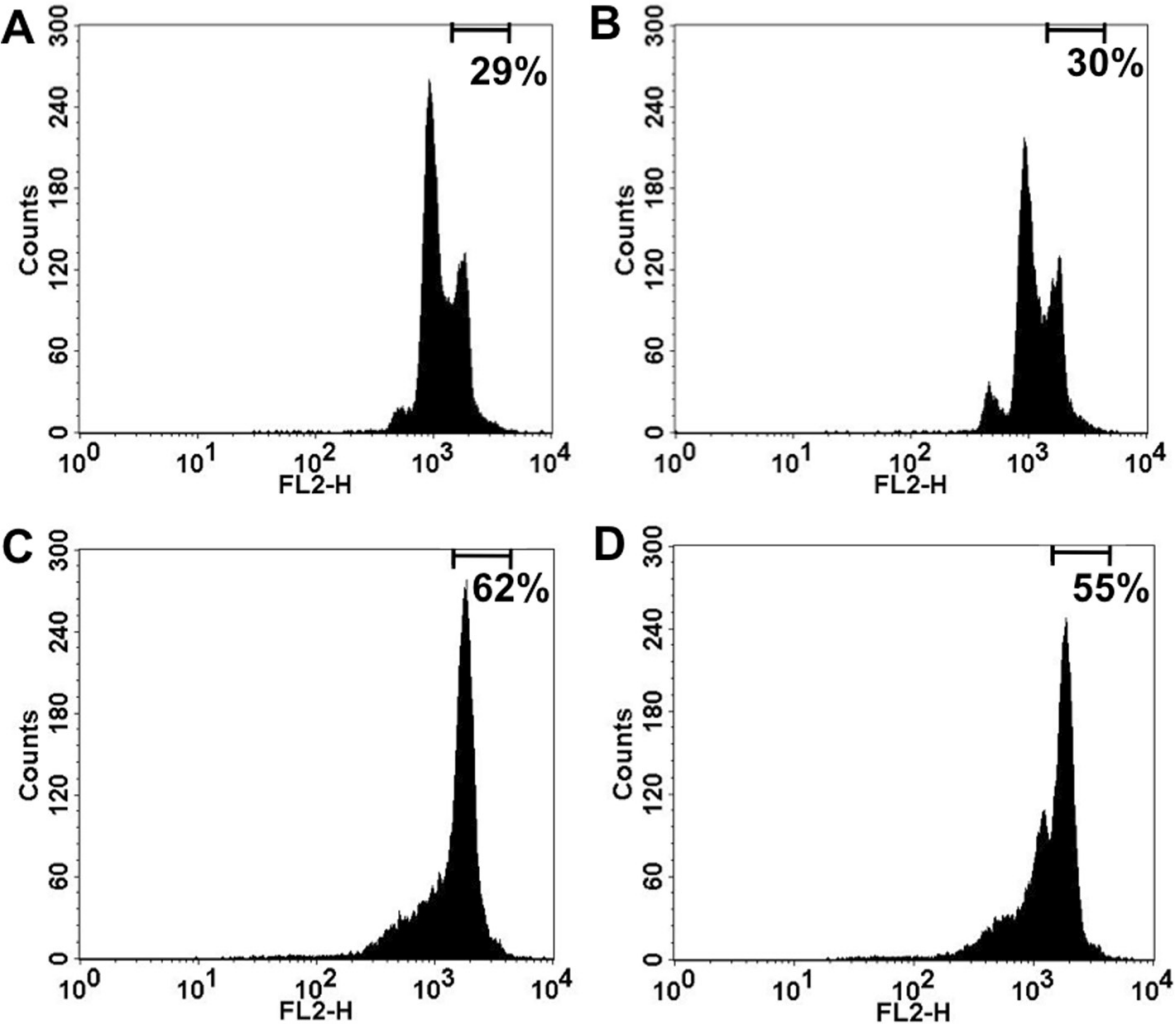
Analysis of the G2/M phase after incubation with temozolomide. Representative registrations of the flow cytometric analysis of neuroblastoma (SHEP) cells after staining with propidium iodide (48 h). The percentage of cells with a diploid DNA content indicating the G2/M phase is indicated in the upper right corner. Panel **a** control; Panel **b** temozolomide 10 μM; Panel **c** temozolomide 100 μM, Panel **d** temozolomide 100 μM with pretreatment with midazolam (16 μM; 6 h)

**Fig. 6:**
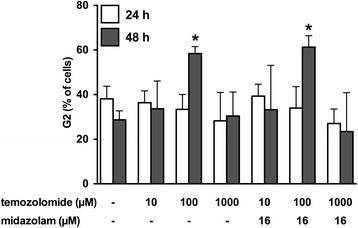
Effect of pretreatment with midazolam to the G2/M arrest induced by temozolomide. The cell cycle was analysed by flow cytometric measurement after staining of cells with propidium iodide. The percentage of cells with a diploid DNA content indicating the G2/M phase of the cell cycle was detected to discriminate the expected effect of temozolomide. Whereas no G2/M arrest was detected after 24 h, temozolomide (100 μM) induced a significant increase of G2/M positive cells after 48 h. This effect was not attenuated by pretreatment with midazolam (16 μM) (*n* = 5; data are expressed as mean ± SD; * = *P* < 0.05)

The flow cytometric measurement of propidium iodide-stained DNA enables to discriminate different contents of intracellular DNA in order to specify the cell cycle. We investigated the impact of a wide range of concentrations of TMZ (10, 100, 1000 μM) on the fraction of cells with a diploid DNA content indicating the G2/M phase. No difference was detected with or without pretreatment of midazolam (16 μM) after incubation for 24 h. After 48 h, the fraction of cells being in the G2/M phase was significantly increased after treatment with TMZ (100 μM), while pretreatment with midazolam (16 μM) did not modify this effect (Figs. 5 and 6).

## Discussion

In the present study, we investigated the dose-dependent effects of midazolam on cell viability in neuroblastoma (SHEP) cells and the impact of midazolam pretreatment and coapplication on the cytotoxicity and antiproliferative effects of TMZ. Furthermore, the effect on the main anti-cancer mechanism of TMZ, the G2/M arrest, was evaluated.

We revealed that midazolam alone induced a significant increase of cell viability with 2, 4, 8 and 16 μM, whereas 128, 256 and 512 μM of midazolam reduced cell viability (IC_50_: 227 μM). Transition from protective to toxic effects of midazolam was estimated to occur at 79.4 μM. When administered as a pretreatment 6 h prior to TMZ exposure, midazolam at a stimulatory concentration of 16 μM significantly increased the IC_25_ and IC_50_ of TMZ, whereas the IC_75_ remained unaffected. Pretreatment followed by coincubation abolished this effect and lowered the IC_75_ of TMZ as a sign of additional toxicity. The antiproliferative effect of TMZ (1000 μM) was significantly attenuated by pretreatment with midazolam, whereas pretreatment followed by coincubation with midazolam had no impact. With view to cell cycle analysis, TMZ (100 μM) increased the fraction of cells in G2/M phase after 48 h. Pretreatment followed by coincubation with midazolam did not affect the G2/M arrest.

To date, data focusing on a pharmacologic interference between the sedative midazolam and the chemotherapeutic agent TMZ were lacking. Since neuroblastoma tumour cells are a common therapeutic target of TMZ in clinical practice, we chose an *in vitro* model of a human neuroblastoma cell line. Furthermore, the comparatively high incidence of this tumour in childhood increases the probability of midazolam being co-applied to TMZ, therefore possibly increasing clinical relevance of the supposed effect on the cytotoxic potency of TMZ. However, to mirror clinical practice and issues with an *in vitro* study has multiple limitations in particular with view to pharmacological parameters like half-life (t_1/2_), peak levels, renal and hepatic elimination and tissue concentration. Nevertheless, this study reveales first evidence, that midazolam ameliorates the cytotoxic effects of TMZ in neuroblastoma cells *in vitro*. The time period of pretreatment with midazolam was 6 h in our study and therefore reflects an increased t_1/2_ of midazolam in children and infants compared to adults [18]. As hepatic or renal impairment and mode of administration strongly alters t_1/2_, a single time period of pretreatment might not be sufficient to investigate a so far unknown effect. Further evaluation is necessary to specify the pharmacokinetic impact on the cytoprotective effects of midazolam. One of the most frequently discussed criticisms of pharmacologic *in vitro* studies involves the concentrations of the applied substances, as they frequently differ from plasma or tissue concentrations used in clinical practice. In our study, we used a broad spectrum of concentrations of midazolam and temozolomide. The logarithmic increase of the applied concentrations enabled us to discriminate small effects of low concentrations of midazolam and the evaluation of the IC_25_, IC_50_ and IC_75_ of temozolomide. The concentration of midazolam (16 μM) used for pretreatment in our study was within the concentration range reached after premedication and continuous sedation (0.3 - 23 μM) as reported previously [19–21]. Nevertheless, a comparison of *in vitro* and *in vivo* concentrations of midazolam remains somewhat artificial. Three concentrations of TMZ were tested with view to an expected G2/M-arrest. Solely 100 μM of TMZ induced this effect after 48 h. This concentration is comparable to plasma levels of patients treated with temozolomide (72 μM), but is 10-fold higher compared to levels of TMZ in the cerebrospinal fluid of these patients [22]. However, effective concentrations in targeted tissues remain unclear and further investigations may be required to characterise the impact of TMZ at different tissue concentrations and time periods of treatment.

The observed increase of cell viability in a neuroblastoma cell line after incubation with low concentrations of midazolam is a counterintuitive finding. Sedatives like midazolam are known as potentially harmful agents especially for neuronal cells with apoptosis-inducing properties at high concentrations, as described above. Previously, Chong and colleagues had shown that midazolam is capable of protecting against reactive oxygen species (ROS) induced cell death in B35 neuroblastoma cells [23]. They reported that pretreatment with midazolam leads to protection against ROS by induction of Akt phosphorylation after activation of phosphoinositol-3-kinase (PI3K). Interestingly, the pretreatment with midazolam was comparable to our study design with regard to treatment duration (8 h) and applied concentrations of midazolam (5 and 10 μM). While their data indicate that incubation with midazolam alone induces Akt phosphorylation, it remains unknown, whether this leads to increased cell viability also in the absence of ROS and, if so, this effect could be abolished by blocking the phosphoinositol-3-kinase. Thus, it remains an open question if Akt activation is involved in the viability enhancing effect of midazolam in our present study. Another study revealed, that midazolam (10 μM) attenuates the antiproliferative effect of glucose oxygen deprivation (GOD) by modulating the profile of pro- and antiapoptotic proteins in astrocytes [24]. As in the study by Chong et al. [23] however, again no data were presented regarding the impact of midazolam alone on cell viability. Guo and co-workers reported cytoprotective effects of midazolam (0.4-40 μM) due to stimulation of steroidogenesis after corticosterone-induced toxicity in rat astrocytes [25]. Midazolam induced the release of pregnenolone and progesterone into the medium, while inhibition of pregnenolone metabolism abolished the protective effect of midazolam. To summarise, Akt phosporylation, modulation of apoptosis-regulating proteins and stimulation of steroidogenesis have been associated with cytoprotective effects of midazolam, although a positive effect like an increase of cell proliferation and viability in the absence of a toxic stimulus has not been reported. Therefore, the potential role of these mechanisms for the protective properties described in our study remains unclear. Whereas only low concentrations of midazolam were investigated in those studies, we evaluated a broader concentration range. This approach enabled us to observe a dose–response relationship phenomenon for midazolam, which is known as hormesis. Hormesis is a toxicological concept, which is defined by Kendig et al. as a “dose–response relationship for a single endpoint that is characterised by reversal of response between low and high doses of chemicals, biological molecules, physical stressors, or any other initiators of a response” [17]. We observed the typical inverted u-shaped dose response curve, which indicates a hormetic response of neuroblastoma cells after incubation with midazolam and confirms a dose-dependent stimulatory and inhibitory effect of this agent. There is considerable evidence, that many endogenous mediators, drugs and toxines can induce hormesis via receptor- and cell signaling mechanisms, e.g. the PI3K, ERK1/2 and p38-pathway [26]. Further investigations are required to determine which mechanisms are involved in the hormetic response induced by midazolam. In contrast, TMZ did not induce a hormetic effect in neuroblastoma cells in our study, which is in line with previous results. Previous studies investigating concentrations of 0.1-10 μM TMZ revealed an anti-cancer effect without any evidence for hormesis [27–29]. Another, more methodological reason could be the lack of very subtle graduation of low dose concentrations. However, with view to the IC_50_ of both agents, the range of stimulatory concentrations of midazolam (1.7-14 % of IC_50_) was comparable with analysed concentrations of TMZ, which did not induce any stimulatory effect. Taken together, there is no evidence that TMZ is involved in effects based on hormesis [22]. Interestingly, the time course of midazolam application has a major impact on the protective effect. Pretreatment induced a significant increase of the IC_25_ and IC_50_ of TMZ, whereas pretreatment with subsequent coincubation amplified the toxicity at high concentrations (reduced IC_75_) of TMZ. Taken together, these findings suggest that the attenuating properties of low-dose midazolam for TMZ-induced cytotoxicity are dependent on the timing of exposure and the magnitude of the subsequent toxic stimulus.

Further aspects of TMZ-induced toxicity were revealed by the proliferation assay (BrdU), which indicated dose-dependent inhibition of neuroblastoma cell proliferation as expected. Pretreatment with midazolam attenuated the antiproliferative effect of TMZ (1000 μM), whereas pretreatment with subsequent coincubation had no significant impact on cell proliferation. Thus, pretreatment with midazolam is capable to affect cell characteristics even followed by exposure to high TMZ concentrations.

As the anticancer properties of TMZ are primarily related to the induction of a G2 arrest of the cells, we investigated the influence of TMZ with and without midazolam on the cell cycle. Surprisingly, 24 h of incubation with TMZ (10, 100, 1000 μM) did not lead to a significant increase of G2/M-positive cells. The detection of an effect on the fraction of G2/M-positive cells after incubation with TMZ might require an appropriate amount of cell division, which is only reached after 48 h. This hypothesis is based on the result of cell cycle analysis after 48 h, as TMZ (100 μM) increased the fraction of G2/M-positive cells significantly. Pretreatment with midazolam did not alter this result, suggesting a different mode-of-action for midazolam-induced cytoprotection. Although midazolam pretreatment does not seem to influence the specific anticancer mechanisms of TMZ, the increase of mitochondrial activity, as indicated by XTT assay analysis, may contribute to the cytoprotective properties of midazolam. This fits well to the detected hormetic effect of midazolam, as hormesis is generally understood not to be based on a single mechanistic pathway, but rather reflects a complex pattern of cellular reactions to an unspecific sublethal stimulus.

## Conclusion

In this *in vitro* study in neuroblastoma (SHEP) cells, midazolam induced a biphasic action with increased cell viability after incubation at low concentrations, whereas high concentrations led to a profound decline in cell viability. Pretreatment with low concentrations of midazolam attenuated the toxic effect of TMZ, whereas pretreatment followed by coincubation had no cytoprotective effect. High concentrations of TMZ abolished the cytoprotection of pretreatment with midazolam. Coincubation with midazolam aggravated the toxicity of high-dose TMZ. The antiproliferative effect of TMZ was attenuated by pretreatment with midazolam. The TMZ-induced G2/M arrest was detected after 48 h of incubation and was not attenuated by pretreatment with midazolam. Our findings may indicate the possibility of reduced anticancer effects of TMZ in patients pretreated with midazolam and therefore justify further investigations into the interaction of chemotherapeutic agents with frequently used comedications like midazolam and other agents with cytotoxic potential.

## Abbreviations

BrdU: Bromodeoxyuridine
DMSO: Dimethyl sulfoxide
DNA: Deoxyribonucleic acid
EDTA: Ethylenediaminetetraacetic acid
GOD: Glucose oxygen deprivation
IC: Inhibitory concentration
ROS: Reactive oxygen species
RPMI: Roswell Park Memorial Institute
TMZ: Temozolomide
XTT: 2,3-Bis(2-methoxy-4-nitro-5-sulfonyl)-2H-tetrazolium-5-carboxanilide
ZEP: Zero Equivalent Point

## References

[1] Armstrong AE, Dargart J, Reichek J, Walterhouse DO, Matossian D, Cohn RA (2014). Irinotecan and temozolomide for treatment of neuroblastoma in a patient with renal failure on hemodialysis. Pediatr Blood Cancer.

[2] Bagatell R, London WB, Wagner LM, Voss SD, Stewart CF, Maris JM (2011). Phase II study of irinotecan and temozolomide in children with relapsed or refractory neuroblastoma: a Children’s Oncology Group study. J Clin Oncol.

[3] Bagatell R, Norris R, Ingle AM, Ahern C, Voss S, Fox E (2014). Phase 1 trial of temsirolimus in combination with irinotecan and temozolomide in children, adolescents and young adults with relapsed or refractory solid tumors: a Children’s Oncology Group Study. Pediatr Blood Cancer.

[4] De Sio L, Milano GM, Castellano A, Jenkner A, Fidani P, Dominici C (2006). Temozolomide in resistant or relapsed pediatric solid tumors. Pediatr Blood Cancer.

[5] Grill J, Geoerger B, Gesner L, Perek D, Leblond P, Cañete A (2013). European Consortium Innovative Therapies for Children with Cancer (ITCC) and the European Society for Paediatric Oncology (SIOPE) brain tumor group: Phase II study of irinotecan in combination with temozolomide (TEMIRI) in children with recurrent or refractory medulloblastoma: a joint ITCC and SIOPE brain tumor study. Neuro Oncol.

[6] Middlemas DS, Stewart CF, Kirstein MN, Poquette C, Friedman HS, Houghton PJ (2000). Biochemical correlates of temozolomide sensitivity in pediatric solid tumor xenograft models. Clin Cancer Res.

[7] Rubie H, Chisholm J, Defachelles AS, Morland B, Munzer C, Valteau-Couanet D (2006). Société Françaisedes Cancers de l’Enfant, United Kingdom Children Cancer Study Group-New Agents Group Study: Phase II study of temozolomide in relapsed or refractory high-risk neuroblastoma: a joint Société Française des Cancers de l’Enfant and United Kingdom Children Cancer Study Group-New Agents Group Study. J Clin Oncol.

[8] Filippi-Chiela EC, Thomé MP, Bueno E, Silva MM, Pelegrini AL, Ledur PF (2013). Resveratrol abrogates the Temozolomide-induced G2 arrest leading to mitotic catastrophe and reinforces the Temozolomide-induced senescence in glioma cells. BMC Cancer.

[9] Kain ZN, Mayes LC, Bell C, Weisman S, Hofstadter MB, Rimar S (1997). Premedication in the United States: a status report. Anesth Analg.

[10] Machata A-M, Willschke H, Kabon B, Kettner SC, Marhofer P (2008). Propofol-based sedation regimen for infants and children undergoing ambulatory magnetic resonance imaging. Br J Anaesth.

[11] Barrett JS, Patel D, Dombrowsky E, Bajaj G, Skolnik JM (2013). Risk assessment of drug interaction potential and concomitant dosing pattern on targeted toxicities in pediatric cancer patients. AAPS J.

[12] Stevens MF, Werdehausen R, Gaza N, Hermanns H, Kremer D, Bauer I (2011). Midazolam activates the intrinsic pathway of apoptosis independent of benzodiazepine and death receptor signaling. Reg Anesth Pain Med.

[13] Ohno S, Kobayashi K, Uchida S, Amano O, Sakagami H, Nagasaka H (2012). Cytotoxicity and type of cell death induced by midazolam in human oral normal and tumor cells. Anticancer Res.

[14] Biedler JL, Helson L, Spengler BA (1973). Morphology and growth, tumorigenicity, and cytogenetics of human neuroblastoma cells in continuous culture. Cancer Res.

[15] Fulda S, Susin SA, Kroemer G, Debatin KM (1998). Molecular ordering of apoptosis induced by anticancer drugs in neuroblastoma cells. Cancer Res.

[16] Riccardi C, Nicoletti I (2006). Analysis of apoptosis by propidium iodide staining and flow cytometry. Nat Protoc.

[17] Kendig EL, Le HH, Belcher SM (2010). Defining hormesis: evaluation of a complex concentration response phenomenon. Int J Toxicol.

[18] Pacifici GM (2014). Clinical Pharmacology of Midazolam in Neonates and Children: Effect of Disease-A Review. Int J Pediatr.

[19] Brosius KK, Bannister CF (2003). Midazolam premedication in children: a comparison of two oral dosage formulations on sedation score and plasma midazolam levels. Anesth Analg.

[20] Hartwig S, Roth B, Theisohn M (1991). Clinical experience with continuous intravenous sedation using midazolam and fentanyl in the paediatric intensive care unit. Eur J Pediatr.

[21] Mulla H, McCormack P, Lawson G, Firmin RK, Upton DR (2003). Pharmacokinetics of midazolam in neonates undergoing extracorporeal membrane oxygenation. Anesthesiology.

[22] Ostermann S, Csajka C, Buclin T, Leyvraz S, Lejeune F, Decosterd LA (2004). Plasma and cerebrospinal fluid population pharmacokinetics of temozolomide in malignant glioma patients. Clin Cancer Res.

[23] Chong WS, Hyun CL, Park MK, Park JM, Song H-O, Park T (2012). Midazolam protects B35 neuroblastoma cells through Akt-phosphorylation in reactive oxygen species derived cellular injury. Korean J Anesthesiol.

[24] Liu L, You Q, Tu Y, Li Q, Zheng L, Li X (2015). Midazolam inhibits the apoptosis of astrocytes induced by oxygen glucose deprivation via targeting JAK2-STAT3 signaling pathway. Cell Physiol Biochem.

[25] Guo W-Z, Miao Y-L, An L-N, Wang X-Y, Pan N-L, Ma Y-Q (2013). Midazolam provides cytoprotective effect during corticosterone-induced damages in rat astrocytes by stimulating steroidogenesis. Neurosci Lett.

[26] Calabrese EJ (2013). Hormetic mechanisms. Crit Rev Toxicol.

[27] Jakubowicz-Gil J, Langner E, Bądziul D, Wertel I, Rzeski W (2013). Apoptosis induction in human glioblastoma multiforme T98G cells upon temozolomide and quercetin treatment. Tumour Biol.

[28] Jakubowicz-Gil J, Langner E, Rzeski W (2011). Kinetic studies of the effects of Temodal and quercetin on astrocytoma cells. Pharmacol Rep.

[29] Raymond E, Izbicka E, Soda H, Gerson SL, Dugan M, Von Hoff DD (1997). Activity of temozolomide against human tumor colony-forming units. Clin Cancer Res.

